# Effects of 4-Week Low-Load Resistance Training with Blood Flow Restriction on Muscle Strength and Left Ventricular Function in Young Swimmers: A Pilot Randomized Trial

**DOI:** 10.5114/jhk/163013

**Published:** 2023-07-15

**Authors:** Zhenhuan Wang, Muhammed M. Atakan, Burak Acar, Rui Xiong, Li Peng

**Affiliations:** 1 Key Lab of General Administration of Sport, Southwest University, Chongqing, China.; 2 Institute for Health and Sport, Victoria University, Footscray, Melbourne, Australia.; 3 Division of Exercise Nutrition and Metabolism, Faculty of Sport Sciences, Hacettepe University, Ankara, Turkey.; 4 Department of Cardiology, Faculty of Medicine, Kocaeli University, Kocaeli, Turkey.

**Keywords:** occlusion training, resistance exercise, left ventricular function, muscle strength, young males

## Abstract

Low-load resistance training combined with blood flow restriction (BFR) is known to result in muscle hypertrophy and strength similar to that observed with higher loads. However, the effects of resistance training with BFR on cardiac structure and cardiac function remain largely unknown. Therefore, the purpose of this randomized study was to compare the effects of conventional high-load resistance training (HL-RT) with the effects of low-load resistance training with BFR (LL-BFR) on muscle strength and left ventricular function. Sixteen young swimmers (mean ± standard deviation: age = 19.7 ± 1.6 years, body mass = 78.9 ± 9.7 kg, body height = 180.8 ± 5.8 cm) were randomly allocated to a conventional HL-RT group (n = 8) or a LL-BFR group (n = 8) with a pressure band (200 mmHg) placed on both thighs of participants for 4 weeks (3 days•week^-1^). Outcome measures were taken at baseline and after 4 weeks of training, and included body composition, one-repetition maximum (1RM) back squat, and echocardiography measures. The 1RM back squat significantly improved (partial eta squared (Ƞ^2^) = 0.365; p = 0.013) in HL-RT (mean difference (Δ) = 6.6 kg; [95% confidence interval (CI) −7.09 to 20.27]) and LL-BFR groups (Δ = 14.7 kg; [95% CI 3.39 to 26.10]), with no main effect of group or group × time interaction (p > 0.05). Interventricular septum end-systolic thickness showed a slight but statistically significant increase in LL-BFR and HL-RT groups (Ƞ^2^ = 0.253; p = 0.047), yet there was no main effect of group or group × time interaction (p > 0.05). There were no statistically significant changes (p > 0.05) in other cardiac structure or function parameters (e.g., left ventricular (LV) mass, LV cardiac output, LV ejection fraction, LV stroke volume) after the training programs. Results suggest that 4 weeks of HL-RT and LL-BFR improve muscle strength similarly with limited effects on left ventricular function in young swimmers.

## Introduction

Resistance training is an effective exercise modality for improving muscle strength, hypertrophy, cardiac structure and function, as well as sports performance (Atakan et al., 2021; Cicek et al., 2017; duManoir et al., 2007; Winett and Carpinelli, 2001). To maximize the benefits of RT, in the absence of artificially induced ischemia (i.e., occlusion training), loads in excess of 65–70% of one-repetition maximum (1RM) are needed (McDonagh and Davies, 1984; Schoenfeld, 2010). Furthermore, training at intensities as low as 30% of 1RM is known to result in complete motor unit recruitment provided that sets are carried out to muscular failure (Burd et al., 2012; Campos et al., 2002). Additionally, a meta-analysis by Schoenfeld et al. (2016) that investigated the effects of resistance training loads on muscle hypertrophy and strength showed that resistance training with loads ≤50–60% of 1RM could promote substantial increases in muscle strength and hypertrophy in untrained individuals. However, there is a strong trend for heavier load conditions to be superior in terms of strength and hypertrophy outcomes (Lopez et al., 2021; Refalo et al., 2021; Schoenfeld et al., 2016). From this point of view, improvements in muscular strength, hypertrophy, and muscular endurance seem to be load-dependent, with superior increases in muscle strength following high-load resistance training (HL-RT) programs. However, heavy-load resistance exercise is often challenging and not attainable for certain individuals, such as those with chronic disease, elderly individuals, and rehabilitating and recovering athletes.

Resistance exercise at low-loads, *e.g.*, 20–40% of 1RM, combined with blood flow restriction (BFR) is a novel exercise modality applied by partially restricting the arterial inflow and fully restricting the venous outflow in working musculature during exercise (de Mendonca et al., 2022; Picon-Martinez et al., 2021; Volga Fernandes et al., 2022). Indeed, resistance exercise with BFR stimulates similar muscular adaptations as high-load exercise (Centner et al., 2022; Scott et al., 2015, 2018; Suga et al., 2012; Takarada et al., 2002), such as depressed pro-inflammatory proteins (Deus et al., 2022), increased muscle mass, strength, and cross-sectional area in humans (Akkoç and Gözübüyük, 2019; Hughes et al., 2017; Takarada et al., 2000; Wilk et al., 2020), likely through stimulating similar cellular and molecular pathways (Davids et al., 2021). These findings make low-load resistance exercise with BFR a surrogate for lifting heavy weights in order to obtain hypertrophy and muscle strength. However, despite mounting evidence for the general efficacy of low-load resistance exercise with BFR on muscular strength, muscular hypertrophy, and muscular endurance in different populations, such as non-athletes (Loenneke et al., 2012; May et al., 2022; Patterson et al., 2019; Pignanelli et al., 2020), lower-limb injured adults (Ladlow et al., 2018), those with Parkinson's disease (Douris et al., 2022), older adults (mean age = 69.4 years) (Douris et al., 2022), cardiovascular surgery patients (Ogawa et al., 2021), and athletes (Scott et al., 2016; Wortman et al., 2021), there has been little implementation of this exercise modality to determine its effects on cardiac structure and cardiac function (Kambič et al., 2022).

It is well documented that physiological, morphological, neuromuscular, biomechanical and technical factors play an important role in swimming performance (Crowley et al., 2017). The current evidence suggests that there is a positive association of lower body strength and specifically, upper limb muscle strength with swimming performance (Smith et al., 2002; West et al., 2011). However, while resistance training combined with BFR was incorporated into training for various types of athletes (Patterson et al., 2019), to the best of our knowledge, no study to date has investigated the effects of this exercise modality on swimmers. Moreover, given the importance of dry land training programs for swimmers, it needs to be determined whether resistance training with BFR would be useful to improve muscle strength in swimmers.

Endurance and resistance training might provide a substantial stimulus to elicit significant improvements in myocardial structure and function, particularly in the left ventricle (LV) (Fleck et al., 1993), which is altogether considered the athlete’s heart (Pelliccia, 2000). Such benefits of resistance training are dependent on various factors, such as training intensity, the type and duration, genetic factors, age, gender, size, ethnicity, and sporting discipline (Haykowsky et al., 2007). However, the effects of low-load resistance training with BFR (LL-BFR) on cardiac structure and cardiac functions remain elusive. Of the limited studies available, Kambič et al. (2019) showed that 8 weeks of supervised biweekly LL-BFR involving unilateral knee extension at 30–40% of 1RM did not change the LV ejection fraction (LVEF) in those with coronary artery disease. In another study by the same group (Kambič et al., 2021), it was reported that 8 weeks of BRF combined with resistance training involving 30%–40%1RM unilateral knee extension improved systolic blood pressure. It is worth noting that considering the nature of the exercise protocols applied in the previous studies (Kambič et al., 2019, 2021; Zhao et al., 2021), which were mainly based on the contractions of limited muscle groups, it is very unlikely that the training regimens of these studies would elicit a sufficient stimulus for remarkable changes in cardiac structure and functions in humans. Hence, it is presently unknown whether LL-BFR involving the activation of large muscle groups can lead to more remarkable changes in cardiac structure and cardiac function parameters. Therefore, the aim of this pilot study was to investigate the effects of 4 weeks of a LL-BFR program involving back squat resistance exercise, which is a compound exercise method that activates muscle groups throughout the lower body as well as core muscles involving the rectus abdominis, obliques, transverse abdominis, and erector spinae, on strength, cardiac structure and function in young swimmers. We hypothesized that in young swimmers, 4 weeks of LL-BFR intervention would elicit similar improvements in strength, cardiac structure and function to a HL-RT program, with even lower mechanical loads.

## Methods

### Research Design and Participants

**Figure 1:**
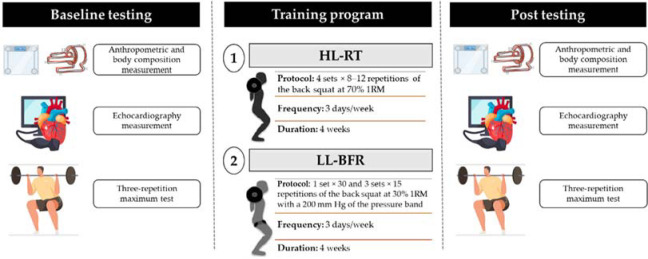
Study design. High-load resistance training (HL-RT), low-load resistance training with blood flow restriction (LL-BFR), and one-repetition maximum (1RM)

This study was a randomized pilot trial. Sixteen young, male swimmers aged 19.7 (±1.6) with resistance training experience from local and university swimming teams volunteered for the study. Inclusion criteria were being healthy, body mass index (BMI) less than 30 kg/m^2^, nonsmokers, free of cardiac disease, and medication/supplement use which might affect the study outcomes. After the baseline assessment, participants were randomly assigned to one of the two training groups: (i) LL-BFR (30% of 1RM) with a pressure band (200 mmHg) placed on both thighs of participants, and (ii) conventional HL-RT (70% of 1RM). Outcome measurements including body composition, 1RM and transthoracic echocardiography were assessed at baseline and 3 days after the final training session (Figure 1). Participants were instructed to refrain from alcohol and caffeine consumption, maintain their habits during the study, and not to engage in any systematic exercise program, including endurance, sprint, and resistance, except for the training programs applied in the present study. Prior to the experiment, all participants were adequately informed of the aims, methods, possible benefits and potential risks of the study as well as any other relevant aspects of the study, and then signed informed consent statements. All experimental protocols and procedures were approved by the Review Board of the Southwest University. The study conformed to the ethical norms and standards outlined in the Declaration of Helsinki.

### Anthropometry and Body Composition

Anthropometry and body composition of participants were assessed before and after the intervention program. Body mass and height were measured using a validated scale (InBody 3.0, Biospace Co., Ltd., Seoul, Korea) and a stadiometer (InBody BSM370, Biospace Co., Ltd., Seoul, Korea) without shoes and with light clothing. Subsequently, the BMI was calculated as weight divided by height squared. After a 10-hour fast and voiding, body composition of participants including total body mass (kg), fat mass (kg), percent fat mass (%), lean mass (kg), and percent lean mass (%) was measured using an inbody720 BIA (InBody 720 Body Composition Analyzer, BioSpace Co., Ltd., Seoul, Korea) according to the manufacturer’s recommendations.

### Echocardiography

To evaluate the effects of the training programs on the LV function, echocardiographic measurements were acquired in the left lateral decubitus position using a Samsung HM70A ultrasound system (Samsung Medison, HM70A, Seoul, South Korea) with a 1.0–4.0 MHz phased array transducer. The measurements were performed in accordance with standard images and techniques recommended in the European Association of Echocardiography and the American Society of Echocardiography's guidelines (Lang et al., 2015). Parameters that were collected with the echocardiographic measurements were: aortic root diameter (AO), left atrial antero-posterior diameter (LAD), LV internal dimension diastole (LVIDd), LV internal dimension systole (LVIDs), LV end-systolic posterior wall thickness (LVPWs), LV end-diastolic posterior wall thickness (LVPWd), interventricular septum end-systolic thickness (IVSs), interventricular septum end-diastolic thickness (IVSd), LV mass (LVM), LVEF, LV fractional shortening (LVFS), LVSV, and LVCO values. LVM and LVFS were calculated with linear measurements obtained from the parasternal long-axis view using the cube formula- Devereux correction. LV volumes and LVEF were calculated with the 2D biplane method (Modified Simpson’s rule). Heart rates (HRs) were noted, and systolic blood pressure (SBP) and diastolic blood pressure (DBP) were also measured using an electronic sphygmomanometer (HEM-8612, OMRON, Kyoto, Japan). Cardiac output was calculated as SV × HR. All measurements were performed three times and the results were averaged for each participant by an experienced cardiologist blinded to the participants’ assignment.

### Three-Repetition Maximum Test

For the determination of the exercise load (%) as well as the effects of training on strength, each participant completed two 3RM back squat tests. Prior to the tests, participants were thoroughly informed of the technical aspects of exercise execution. The warm-up consisted of 1000 m of jogging on a treadmill at 8 km/h and 4 to 10 back squat repetitions at 50% of the perceived maximum load based on previous experience. After 5 min of rest, the test was started at an initial load of 80 to 100% of the perceived maximum load for each participant, and they were asked to perform the maximum number of repetitions until failure. If the number of repetitions was >10, the load was gradually increased by 10% until participants could not perform more than 3 repetitions of the back squat at a certain resistance. A 5-min rest interval was allowed before the next attempt. 1RM was calculated according to the mathematical model proposed by Baechle and Groove (2019), i.e., {1RM = load × ([0.0375 × reps] + 0.978)}. Participants were instructed to refrain from heavy exercise for 48 hours before the evaluation and all tests were continuously monitored to ensure the quality of the data. The pre and post 3RM tests were applied at the same time of the day.

### Training Intervention

Individuals in LL-BFR and HL-RT groups participated in supervised isotonic resistance training programs for 4 weeks (3 days•week^-1^). The training program in the LL-BFR group involved a common and frequently used set and repetition scheme (Patterson et al., 2019) that consisted of four sets of back squat exercises at 30% 1RM, with 30 repetitions in the first set and 15 repetitions in the following three sets, with a pressure band (Beijing Pukang Sport & Medical Co., Ltd, Beijing, China) placed on both thighs of participants (inguinal fold region). Each set was separated by one-minute recovery in the absence of the pressure band. The cuff pressure applied during the back squat repetitions was 200 mm Hg. The cuff material was nylon and 6 cm in width (BStrong, Park City, Utah, USA). The HL-RT group performed 4 sets × 8–12 repetitions of back squat exercises at 70% of the estimated 1RM, interspersed with 3-min rest intervals, in the absence of a pressure band. The training intensity remained constant throughout the training period for both groups and participants were instructed not to perform any physical exercise, external to the program, during the training period.

### Reproducibility of Echocardiography Measurements

Two echocardiography measurements, separated by at least 72 hours, on 10 healthy, young males who did not participate in the main study were performed in accordance with standard images and techniques recommended in the European Association of Echocardiography and the American Society of Echocardiography's guidelines (Lang et al., 2015). The coefficient of variation, intraclass correlation coefficient, and standard error of measurement calculated for AO, LAD, LVIDs, LVIDd, LVPWs, LVPWd, IVSs, IVSd, LVM, LVEF, LVFS, LVSV, and LVCO are presented in Table 1.

**Table 1:** Reproducibility data for cardiac structure and cardiac function parameters (n = 10).

	CV	ICC	SEm
Aortic root diameter (mm)	8.88	0.964	1.19
Left atrial diameter (mm)	6.52	0.889	0.80
LV internal dimension systole (mm)	6.32	0.945	0.86
LV internal dimension diastole (mm)	5.05	0.819	1.1
LV end-systolic posterior wall thickness (mm)	9.67	0.709	0.48
LV end-diastolic posterior wall thickness (mm)	11.58	0.910	0.52
Interventricular septum end-systolic thickness (mm)	13.02	0.850	0.73
Interventricular septum end-diastolic thickness (mm)	10.70	0.664	0.47
LV mass (g)	9.40	0.966	5.63
LV ejection fraction (%)	6.37	0.894	1.84
LV fractional shortening (%)	5.93	0.859	1.01
LV stroke volume (mL)	9.72	0.901	2.92
LV cardiac output (L/min)	12.09	0.833	0.33

Coefficient of variation (CV), intraclass correlation coefficient (ICC), standard error of measurement (SEm), and left ventricle (LV).

### Statistical Analysis

The normality of data distribution was confirmed with the Shapiro-Wilk test. When the data satisfied the normality assumption, the results of the 1RM back squat, cardiac function and cardiac structure parameters were analyzed using two-way analysis of variance (ANOVA) for repeated-measures to determine main effects of group, time, and group × time interaction. The source of explained variance on repeated measure ANOVA was estimated by partial eta squared (Ƞ^2^). A Greenhouse–Geisser correction was applied when the Mauchly’s test of sphericity was violated. Considering that our small sample size did not allow for definitive analysis, this statistical analysis of the data was exploratory only. Effect sizes (*d*) were calculated using Cohen’s *d* method (Cohen, 1988). All data are presented as mean ± standard deviation (SD), as well as mean difference (Δ) and 95% confidence interval (95% CI). Statistical analyses were performed using SPSS Statistics for Windows, Version 21.0 (IBM Corp., Armonk, NY, USA), and the level of significance was set at *p* < 0.05.

## Results

### Training Fidelity

A total of 22 potential participants meeting our inclusion criteria were invited to take part in the study, with six participants not showing up for baseline testing; thus, a total of 16 young, healthy male swimmers completed the study (age = 19.7 ± 1.6 years). All participants assigned to the HL-RT and LL-BFR groups completed the study. There were no significant differences (*p* > 0.05) between groups in terms of participants’ characteristics or the variables evaluated at the beginning of the study (Table 2). No adverse event was reported during testing or training in either group.

**Table 2:** Participants’ characteristics.

	HL-RT	LL-BFR	*p*
**Age, years**	20.1 (2.0)	19.8 (1.2)	0.77
**Body mass, kg**	81.3 (6.2)	76.5 (13.2)	0.36
**Height, cm**	180.3 (6.4)	181.3 (5.1)	0.73
**Body mass index, kg^2^/m**	25.0 (2.3)	23.0 (3.5)	0.36
**Training experience, years**	11.5 (3.7)	9.7 (3.9)	0.37

High-load resistance training (HL-RT) and low-load resistance training with blood flow restriction (LL-BFR). Values are means (standard deviation) for 8 participants per group.

### Changes in Body Composition

**Figure 2:**
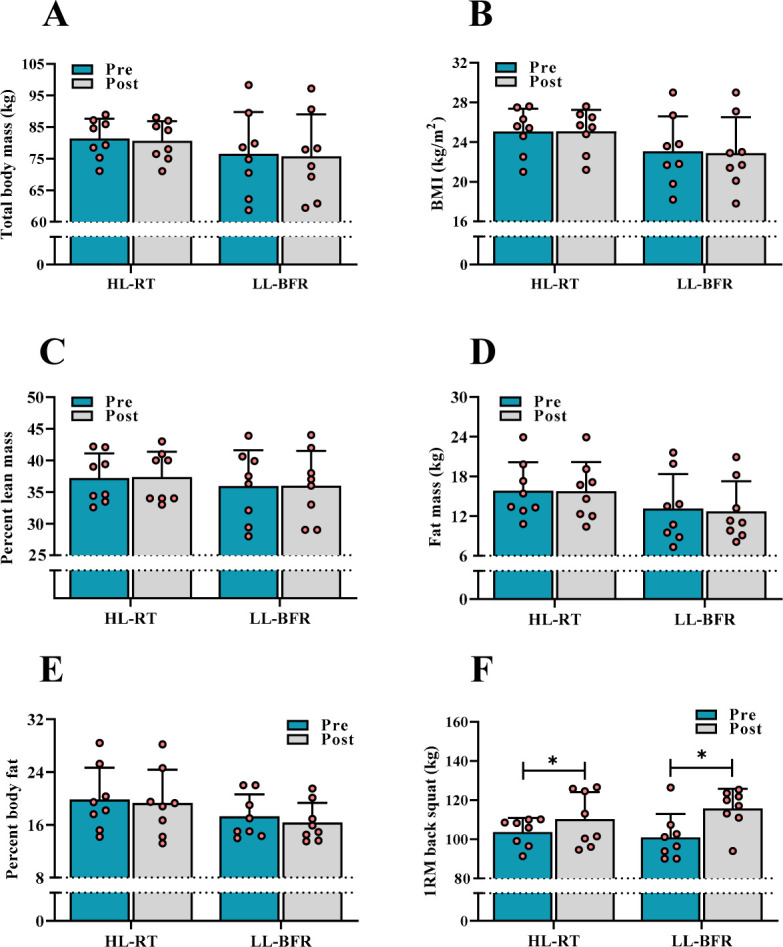
Changes in body composition (A–E) and the 1RM back squat (F) in the HL-RT and LL-BFR groups. *Main effect for condition (p < 0.05), such that the one-repetition maximum (1RM) back squat pre and post are different in both groups. Bars represent means for high-load resistance training (HL-RT) and low-load resistance training with blood flow restriction (LL-BFR) groups with error bars indicating the standard deviation and circles denoting participants. BMI, body mass index

A two-way repeated measures analysis of variance revealed that the HL-RT and LL-BFR programs did not result in a statistically significant change (*p* > 0.05) in total body mass [(HL-RT Δ = −0.763 kg vs. LL-BFR Δ = −0.788 kg); Figure 2A], BMI [(HL-RT Δ = 0.02 kg/m^2^ vs. LL-BFR Δ = −0.20 kg/m^2^); Figure 2B], percent lean mass [(HL-RT Δ = 0.150% vs. LL-BFR Δ = 0.038%); Figure 2C], fat mass [(HL-RT Δ = −0.07 kg vs. LL-BFR Δ = −0.44 kg); Figure 2D] or percent body fat [(HL-RT Δ = −0.13% vs. LL-BFR Δ = −1.03%); Figure 2E].

### Changes in the 1RM Back Squat

There was a significant [F(8.055), Ƞ^2^ = 0.365; *p* = 0.013] increment for the 1RM back squat in the HL-RT (Δ = 6.6 kg; [95% CI −7.09 to 20.27]; *d* = 0.34) and LL-BFR groups (Δ = 14.7 kg; [95% CI 3.39 to 26.10]; *d* = 1.10) (Figure 2F), with no main effect of group or group × time interaction (*p* > 0.05), indicating that both training programs resulted in similar changes in the 1RM back squat.

### Changes in Cardiac Structure and Cardiac Function Parameters

There were no statistically significant differences (*p* > 0.05) in cardiac structure or cardiac function parameters in response to the HL-RT and LL-BFR programs, including AO, LAD, LVIDs, LVIDd, LVPWs, LVPWd, or IVSd, resting SBP, resting DBP, resting HR, LVEF, LVFS and LVSV (Table 3). A significant increase in IVSs was evident in both groups [F(4.736), Ƞ^2^ = 0.253; *p* = 0.047], with no main effect of group or group × time interaction (*p* > 0.05; Table 3). There was a slight trend in LVM [F(3.147), Ƞ^2^ = 0.184; *p* = 0.09] and in LVCO [F(3.293), Ƞ^2^ = 0.190; *p* = 0.09] to be higher following the training interventions, but these differences did not reach statistical significance (*p* > 0.05; Table 3).

**Table 3:** Changes in left ventricular parameters in HL-RT and LL-BFR groups.

Left ventricular parameters	Before	After	Δ	*d*	95% CI
**Aortic root diameter (mm)**					
HL-RT	28.6 ± 2.0	28.9 ± 2.2	0.31	0.42	−0.30 to 0.92
LL-BFR	29.2 ± 2.2	28.6 ± 2.2	−0.61	−0.56	−1.52 to 0.30
**Left atrial diameter (mm)**					
HL-RT	29.4 ± 3.7	30.3 ± 4.8	0.90	0.38	−1.09 to 2.89
LL-BFR	27.7 ± 2.8	28.6 ± 2.8	0.85	0.44	−0.75 to 2.45
**LV internal dimension systole (mm)**					
HL-RT	26.4 ± 5.3	28.6 ± 5.9	2.26	0.36	−2.96 to 7.48
LL-BFR	26.3 ± 8.1	26.0 ± 5.4	−0.26	−0.07	−3.64 to 3.12
**LV internal dimension diastole (mm)**					
HL-RT	49.3 ± 4.8	51.5 ± 6.8	2.22	0.41	−2.31 to 6.77
LL-BFR	49.4 ± 5.0	50.5 ± 5.2	0.66	0.20	−2.12 to 3.44
**LV end-systolic posterior wall thickness (mm)**					
HL-RT	18.0 ± 3.0	17.9 ± 2.2	−0.72	−0.41	−2.20 to 0.75
LL-BFR	18.5 ± 2.4	19.0 ± 3.0	0.48	0.75	−0.05 to 1.03
**LV end-diastolic posterior wall thickness (mm)**					
HL-RT	10.8 ± 1.6	10.6 ± 1.2	−0.05	−0.11	−0.44 to 0.34
LL-BFR	11.2 ± 3.4	11.6 ± 3.3	0.39	0.61	−0.14 to 0.92
**Interventricular septum end-systolic thickness (mm)**					
HL-RT	15.5 ± 2.1	16.0 ± 2.2	0.43	0.52	−0.26 to 1.11
LL-BFR	15.3 ± 3.1	15.8 ± 3.2	0.46	0.57	−0.22 to 1.15
**Interventricular septum end-diastolic thickness (mm)**					
HL-RT	10.5 + 1.2	10.6 ± 1.2	0.04	0.24	−0.10 to 0.17
LL-BFR	10.3 ± 1.6	10.7 ± 1.7	0.40	0.63	−0.13 to 0.93
**LV mass (g)**					
HL-RT	194.3 ± 24.4	211.1 ± 51.2	16.81	0.46	−13.91 to 47.53
LL-BFR	194.6 ± 90.6	222.3 ± 87.1	27.66	0.47	−23.04 to 75.35
**Resting systolic blood pressure (mm Hg)**					
HL-RT	123.1 ± 8.6	123.1 ± 15.4	0.00	0.00	−7.50 to 7.50
LL-BFR	116.1 ± 13.4	110.0 ± 8.5	6.13	−0.55	−15.47 to 3.22
**Resting diastolic blood pressure (mm Hg)**					
HL-RT	74.5 ± 12.4	77.4 ± 14.0	2.88	0.33	−4.32 to 10.07
LL-BFR	68.8 ± 10.8	68.8 ± 5.6	0.38	0.04	−7.17 to 7.91
**Resting heart rate (bpm)**					
HL-RT	55.3 ± 5.3	56.0 ± 4.8	0.75	0.65	−0.49 to 1.99
LL-BFR	54.3 ± 4.3	53.6 ± 3.6	−0.63	−0.35	−2.10 to 0.85
**LV ejection fraction (%)**					
HL-RT	71.4 ± 16.7	78.6 ± 8.7	7.25	0.38	−8.64 to 23.15
LL-BFR	77.4 ± 7.9	75.4 ± 6.6	−2.06	−0.29	−8.02 to 3.90
**LV fractional shortening**					
HL-RT	46.7 ± 7.1	44.7 ± 5.8	−2.03	−0.33	−5.23 to 3.17
LL-BFR	47.9 ± 11.4	48.4 ± 9.3	0.51	0.07	−5.93 to 6.95
**LV stroke volume (mL)**					
HL-RT	89.0 ± 17.3	96.5 ± 24.6	7.50	0.48	−5.68 to 20.68
LL-BFR	76.2 ± 31.7	96.1 ± 25.4	19.91	0.43	−18.76 to 58.58
**LV cardiac output (L/min)**					
HL-RT	5.9 ± 1.8	6.0 ± 1.0	0.12	0.06	−1.62 to 1.87
LL-BFR	4.7 ± 1.3	6.0 ± 1.4	1.35	1.41	0.55 to 2.15

Cohen’s effect size (d), confidence interval (CI), delta values (Δ), high-load resistance training (HL-RT), left ventricle (LV), and low-load resistance training with blood flow restriction (LL-BFR). Values are means ± standard deviation for 8 participants per group.

## Discussion

This pilot study is the first to investigate the impact of LL-BFR on strength and the left ventricular function in young swimmers. Results show that in young swimmers, four weeks of LL-BFR and HL-RT resulted in similar significant improvements in the 1RM back squat, with limited effects on the cardiac function and cardiac structure.

### Effects of LL-BFR and HL-RT on the Left Ventricular Function

The magnitude of changes in the central cardiovascular response to exercise with BFR is predominately associated with the level of BFR (Rossow et al., 2012), the mode of exercise (i.e., resistance vs. aerobic) (Staunton et al., 2015), and the mode of application (i.e., continuous vs. intermittent BFR) (Brandner et al., 2015). It was noted that acute resistance exercise combined with BFR could affect central hemodynamic parameters including SBP, DBP, HR, SV, CO and total peripheral vascular resistance (Libardi et al., 2017; May et al., 2017; Staunton et al., 2015). However, it has yet to be fully explored whether these acute responses would lead to long-term cardiovascular adaptations (Wong et al., 2021). In the present study, we found that four weeks of LL-BFR, involving back squat resistance exercise activating larger muscle groups than those in the previous research, did not change the cardiac function and cardiac structure in young, healthy swimmers. Similar to our findings, Zhao et al. (2021) found that the cardiac function parameters, such as LVEF, LVSV, and LVCO, remained unchanged following eight weeks (5 days•week^-1^) of high- and low-pressure LL-BFR (30% of 1RM) in young, healthy males (Zhao et al., 2021). Similarly, Kambič et al. (2019) reported that LVEF and VL muscle thickness did not change in response to eight weeks of LL-BFR (30–40% of 1RM unilateral knee extension with BFR applied on each thigh using a pneumatic cuff with pressure of 15 and 20 mm Hg) in those with coronary artery disease. In contrast, a recent study by Lu et al. (2020) documented that stroke volume and cardiac output increased to a similar degree after 12 weeks of LL-BFR with 120 and 180 mm Hg occlusion pressure on lower limb muscles in young college students. Further research is needed to shed light on the discrepancy among these studies. Also, resistance training with BFR performed at higher intensities (>50% of 1RM) than applied in the present study may be needed for notable cardiological adaptation. However, it is worth noting that the magnitude of changes in central hemodynamic response is usually lower after resistance exercise with BFR compared to high-load resistance exercise alone, as pointed out in a review by Patterson et al. (2019). Additionally, the present study was likely underpowered to detect statistical differences between groups due to the small sample size (*n* = 8 per group) and short duration of the training programs (four weeks) which did not provide sufficient stimuli. Hence, as highlighted by Voulgari et al. (2013), further research with higher statistical power and longer duration is needed to elucidate the effects of resistance training with BFR on the cardiac structure and function in different populations (i.e., non-athletes, athletes and those with different metabolic diseases). Moreover, according to the theory of Morganroth et al. (1975), athletes engaging in mainly static or isometric exercise (e.g., weightlifting) are likely to exhibit predominantly increased LV wall thickness with unchanged LV chamber size induced by pressure overload accompanying the high systemic arterial pressure during resistance exercise, which in turn results in concentric left ventricular hypertrophy characterized by an increased ratio of wall thickness to radius in strength-trained athletes (Pluim et al., 2000). In this sense, participants of the current study were young swimmers with resistance training experience, who were regularly trained in swimming. The unique aspects of swimming are immersion of the body in water, supine posture, use of both the upper and lower limbs, and the novel requirement of breath holding, resulting in exercise-induced LV remodeling (Currie et al., 2018; Holmér et al., 1974). Also, research reported that regular swimming training leads to exercise-induced LV remodeling as reflected by increases in wall thickness, internal diastolic diameters and the LV mass index (Currie et al., 2018; Venckunas et al., 2008). However, while we cannot categorize our participants as well-trained or elite athletes, it is likely that their training routines did result in improvements in their LV function (Shapiro, 1984; Urhausen and Kindermann, 1999). This might have limited the effects of the training programs applied in the current study and caused that our short-term resistance training programs failed to provide a significant stimulus to improve cardiac structure and function of our participants.

### Effects of the LL-BFR and HL-RT on the 1RM Back Squat

In the present study, for the first time in young swimmers, we found that short-term LL-BFR and HL-RT significantly improved strength in a similar manner as evidenced by improvement in the 1RM back squat. These findings indicate that the use of short-term resistance training with BFR is effective in increasing strength in swimmers without profound effects on the LV function. In agreement with our findings, the prevailing body of research has indicated that resistance exercise with BFR is effective in increasing skeletal muscle strength and/or hypertrophy in healthy young populations (Lixandrão et al., 2018; Loenneke et al., 2012; Slysz et al., 2016). Also, a systematic review by Wortman et al. (2012) documented that resistance exercise with BFR increased strength, muscle size, and markers of sports performance in athletes, which is in line with our findings. Furthermore, Laurentino et al. (2012) reported that eight weeks (×2/wk) of low-load knee extension training at 20% of 1RM increased knee extension 1RM (40.1%) in physically active males. Similarly, it was reported that two weeks of BFR training (×2/day, 6 days/wk) at 20% of 1RM improved the squat 1RM in young individuals (Abe et al., 2005), which concurs with the results of the present study. In support of the above, a recent study by Geng et al. (2022) reported improved muscle strength in national-level para-alpine standing skiers after two weeks of resistance training combined with BFR. Although the exact molecular mechanisms driving the hypertrophic adaptations following LL-BFR are unknown at present, a recent study by Davids and colleagues (2021) showed that this form of resistance training enhanced muscular responses without the need for high mechanical loads likely through stimulating similar cellular and molecular pathways to HL-RT. Furthermore, considering the short-term training programs in the present study and no significant increase in lean mass, it is likely that the observed increase in the 1RM of our participants was due to neural adaptations known to increase strength during the first couple of weeks of training (May et al., 2022; Sale, 1988).

### Limitations of the Study

While this study provides new insight regarding the cardiac responses to short-term resistance training combined with BFR, some important limitations of the experiment should be acknowledged. First, the current study was limited by the small sample size and was likely to be underpowered for the detection of statistical significance, thus the current investigation should be deemed a pilot study. Second, we only recruited healthy, young swimmers and therefore, transference of our results to inactive or clinical populations is unknown at present. Third, given the absence of a control group in the present study that did not participate in any training intervention (i.e., HL-RT, LL-BFR), it remains elusive whether the statistically significant (i.e., 1RM back squat) or non-significant (i.e., AOD, LV mass, LV cardiac output) changes observed in the intervention groups would differ from a non-exercising group. Finally, as highlighted by a recent review (Mazzolari et al., 2022), further studies should consider the use of equivalence and non-inferiority tests for interventional exercise studies similar to the current one. Equivalence and non-inferiority tests are based on a margin using the point-estimate method or the fixed-margin method (Althunian et al., 2017), stating that the non-inferiority margin should not be larger than the smallest effect of the reference intervention (i.e., HL-RT) and only, in this case, can be reliably compared with another intervention (i.e., LL-BFR) (Mazzolari et al., 2022).

## Conclusions

This study shows that four weeks of low-load resistance training with blood flow restriction is an alternative training modality to high-load resistance training that can similarly improve muscle strength, with limited effect on the left ventricular function in young swimmers. Further research with larger sample size and longer duration is needed to determine the dose of low-load resistance training with blood flow restriction necessary to improve cardiac structure and cardiac function.
